# The Antenatal Corticosteroids Trial (ACT): a secondary analysis to explore site differences in a multi-country trial

**DOI:** 10.1186/s12978-016-0179-z

**Published:** 2016-05-24

**Authors:** Karen Klein, Elizabeth M. McClure, Daniela Colaci, Vanessa Thorsten, Patricia L. Hibberd, Fabian Esamai, Ana Garces, Archana Patel, Sarah Saleem, Omrana Pasha, Elwyn Chomba, Waldemar A. Carlo, Nancy F. Krebs, Shivaprasad Goudar, Richard J. Derman, Edward A Liechty, Marion Koso-Thomas, Pierre M. Buekens, José M. Belizán, Robert L. Goldenberg, Fernando Althabe

**Affiliations:** Institute for Clinical Effectiveness and Health Policy (IECS), Buenos Aires, Argentina; RTI International, Durham, NC USA; Massachusetts General Hospital, Boston, MA USA; Moi University School of Medicine, Eldoret, Kenya; Fundación para la Alimentación y Nutrición de Centro América y Panamá, Guatemala City, Guatemala; Lata Medical Research Foundation, Indira Gandhi Government Medical College, Nagpur, India; Department of Community Health Sciences, Aga Khan University, Karachi, Pakistan; University Teaching Hospital, Lusaka, Zambia; University of Alabama at Birmingham, Birmingham, AL USA; University of Colorado School of Medicine, Denver, Colorado USA; KLE University’s Jawaharlal Nehru Medical College, Belgaum, Karnataka India; Christiana Health Care, Newark, DE USA; School of Medicine, Indiana University, Indianapolis, IN USA; Eunice Kennedy Shriver National Institute of Child Health and Human Development, Bethesda, MD USA; Tulane School of Public Health & Tropical Medicine, New Orleans, Louisiana USA; Department of Obstetrics and Gynecology, Columbia University, New York, NY USA

## Abstract

**Background:**

The Antenatal Corticosteroid Trial (ACT) assessed the feasibility, effectiveness, and safety of a multifaceted intervention to increase the use of antenatal corticosteroids (ACS) in mothers at risk of preterm birth at all levels of care in low and middle-income countries. The intervention effectively increased the use of ACS but had no overall impact on neonatal mortality in the targeted <5^th^ percentile birth weight infants. Being in the intervention clusters was also associated with an overall increase in neonatal deaths. We sought to explore plausible pathways through which this intervention increased neonatal mortality.

**Methods:**

We conducted secondary analyses to assess site differences in outcome and potential explanations for the differences in outcomes if found. By site, and in the intervention and control clusters, we evaluated characteristics of the mothers and care systems, the proportion of the <5^th^ percentile infants and the overall population that received ACS, the rates of possible severe bacterial infection (pSBI), determined from clinical signs, and neonatal mortality rates.

**Results:**

There were substantial differences between the sites in both participant and health system characteristics, with Guatemala and Argentina generally having the highest levels of care. In some sites there were substantial differences in the health system characteristics between the intervention and control clusters. The increase in ACS in the intervention clusters was similar among the sites. While overall, there was no difference in neonatal mortality among <5^th^ percentile births between the intervention and control clusters, Guatemala and Pakistan both had significant reductions in neonatal mortality in the <5^th^ percentile infants in the intervention clusters. The improvement in neonatal mortality in the Guatemalan site in the <5^th^ percentile infants was associated with a higher level of care at the site and an improvement in care in the intervention clusters. There was a significant increase overall in neonatal mortality in the intervention clusters compared to the control. Across sites, this increase in neonatal mortality was statistically significant and most apparent in the African sites. This increase in neonatal mortality was accompanied by a significant increase in pSBI in the African sites.

**Conclusions:**

The improvement in neonatal mortality in the Guatemalan site in the <5^th^ percentile infants was associated with a higher level of care and an improvement in care in the intervention clusters. The increase in neonatal mortality in the intervention clusters across all sites was largely driven by the poorer outcomes in the African sites, which also had an increase in pSBI in the intervention clusters. We emphasize that these results come from secondary analyses. Additional prospective studies are needed to assess the effectiveness and safety of ACS on neonatal health in low resource settings.

**Trial registration:**

Trial registration: clinicaltrials.gov (NCT01084096)

## Background

The use of antenatal corticosteroids (ACS) is believed to be among the most effective interventions to reduce neonatal mortality associated with preterm birth. However, the majority of research on its efficacy and safety has been conducted in high-resource settings [1–6]. Few studies on ACS were conducted in low-middle income countries (LMIC) where the overwhelming majority of deaths associated with preterm birth occur [7–9]. Of those studies which have been performed outside of developed countries, several have been in Latin America, with only a few in Asia or Africa and none in the regions of the highest burden of mortality [10–12].

The Global Network Antenatal Corticosteroid Trial (ACT), was a multi-country cluster randomized trial to assess the feasibility, effectiveness, and safety of a multifaceted intervention developed to increase the use of ACS at all levels of health care in LMICs [13, 14]. ACT, which was conducted in seven rural and semi-urban sites in sub-Saharan Africa, Asia and Latin America, showed that the intervention increased ACS use in the intervention clusters (46 % vs.10 % in women with <5th percentile birth weight live births), but it was not associated with a reduction in neonatal mortality in the targeted <5th percentile infants in the intervention clusters. An unexpected finding was that overall neonatal mortality was higher in intervention clusters, with the excess mortality occurring in infants born at >25th percentile site-specific birth weights.

Since ACT was a multi-country intervention, we sought to explore whether regional differences were associated with the impact of the ACS intervention. ACT was pragmatic in design and there was limited data collection beyond the primary study outcomes. Thus, the ability to identify site specific associations is limited. Nonetheless, given that ACT is the largest trial of ACS use in low-income countries and the unanticipated results, in this paper, we further explore neonatal outcomes by site.

## Methods

### Specific aims

The objectives of this secondary analysis were: 1) to describe the effects of the ACT intervention on neonatal mortality, use of ACS, and possible severe neonatal bacterial infection (pSBI) by site; and 2) to assess whether the observed differences were correlated with either the use of ACS, differences in maternal characteristics or in aspects related to care.

### Study design and participants

This is a secondary analysis of data collected during the ACT. The study intervention and methods are described in detail elsewhere [13]. Briefly, the ACT was a two-arm, cluster-randomized trial conducted in seven sites of the Global Network for Women’s and Children’s Health Research (Argentina, Guatemala, Zambia, Kenya, Pakistan, Belgaum (India) and Nagpur (India)). Clusters were organized into strata based on the 28-day neonatal mortality rate by site, and then randomized by the data center (RTI International, Durham, NC, USA). Intervention clusters received a multifaceted intervention (health provider training, posters, pregnancy discs, uterine height tape, and antenatal corticosteroids kits). Providers in the intervention clusters were trained to administer one course of four doses of 6 mg of dexamethasone every 12 h to women identified at high-risk of preterm birth. Along with providers in the formal health systems, traditional birth attendants and community health workers were also trained to identify women at risk and initiate treatment. The intervention period was 18-months, with the start date between 2011 and 2012 varying by site based on the site approval date.

Outcome data were collected by trained registry administrators in a prospective, on-going, population-based maternal and newborn health (MNH) registry independent of the intervention team. The MNH registry was implemented in 2009 [15, 16] so all sites had at least 1 year of pretrial data was available. MNH data were collected during pregnancy, following delivery and at 6-weeks post-partum to determine basic information regarding use of antenatal and delivery care, stillbirth, neonatal mortality and morbidity. Data were collected on observable infant signs of illness which were used to determine possible severe bacterial infection (pSBI) based on the World Health Organization definition, as described in detail elsewhere [Hibberd PL, Hansen NI, Wang M, et al.: Trends in the Incidence of Possible Severe Bacterial Infection and Case Fatality Rates in Rural communities in Sub-Saharan Africa, South Asia and Latin America, 2010–2013: A multicenter prospective cohort study, forthcoming; 17, 18]. In addition, in the intervention clusters, we collected data on women, newborns and intervention process measures.

### Statistical analyses

Generalized linear models were used to evaluate the relationship between covariates and the outcomes of interest (neonatal mortality and pSBI) and to develop point and interval estimates of relative risk (RR) associated with those risk factors. Generalized estimating equations accounted for the correlation of outcomes within cluster to develop appropriate confidence intervals. Analyses were adjusted for randomization strata. Models were log binomial when possible, otherwise Poisson models were utilized. Pre-trial data were analyzed to assess the status of various measures prior to trial implementation. All analyses were done by RTI International with SAS versions 9.3 and 9.4 (SAS Institute, Cary, NC, USA).

## Ethical approvals

The trial was reviewed and approved by the ethics committees at each site. In addition, the World Health Organization and the *Eunice Kennedy Shriver* National Institute of Health and Human Development (NICHD) reviewed and approved the protocol. A Data Monitoring Committee reviewed the study enrollment and safety. All women provided informed consent prior to enrollment.

## Results

A total of 99,742 women were enrolled in ACT across the sites including 62,541 in south Asia, 23,021 in sub-Saharan Africa, and 14,180 in Latin America. Basic maternal characteristics in the sites, as well as in the treatment and control clusters are presented for all sites (Table 1). The percent of women who were age <20 years ranged from 1.8 % in Nagpur, India to 28.8 % in Argentina. Conversely, the percent of women age >35 years of age ranged from 0.2 % in Belgaum, India to 10.4 % in the Kenyan site. Rates of women without any formal education ranged from 2.0 % in Argentina to 80.9 % in the Pakistan site. The highest proportion of women with parity >2 was in Pakistan, where nearly 60 % of women had 2 or more prior pregnancies compared to a low of 11.2 % in the Nagpur site. Within the sites, the maternal demographics were generally similar between the intervention and control clusters.

**Table 1:** Maternal characteristics by site and by intervention (Int) and control (Ctr) clusters^a^

	South Asia (*n* = 62,541)											
	Belgaum, India			Nagpur, India			Pakistan					
	Total	Int	Ctr	Total	Int	Ctr	Total	Int	Ctr			
Women, N	31,530	14,900	16,630	15,095	7,498	7,597	15,916	7,714	8,202			
Maternal age, %												
< 20	8.7	7.2	10.0	1.8	1.6	2.0	3.9	3.7	4.1			
20 - 35	91.1	92.6	89.8	97.9	98.1	97.7	90.1	91.5	88.9			
> 35	0.2	0.2	0.1	0.3	0.3	0.3	5.9	4.8	7.0			
No formal school %	19.3	20.5	18.3	2.5	3.3	1.9	80.9	81.2	80.3			
Parity, %												
0	43.7	43.8	43.6	45.9	45.1	46.8	19.8	19.5	20.1			
1	34.1	35.2	33.8	42.6	43.0	42.4	20.5	20.9	20.2			
2+	21.7	21.0	22.6	11.4	11.9	10.8	59.6	59.6	59.7			
	Sub-Saharan Africa (*n* = 23,021)						Latin America (*n* = 14,180)					
	Zambia			Kenya			Guatemala			Argentina		
	Total	Int	Ctr	Total	Int	Ctr	Total	Int	Ctr	Total	Int	Ctr
Women, N	10,018	4,279	5,739	13,003	5,900	7,103	9,773	5,813	3,960	4,407	2,115	2,292
Maternal age, %												
< 20	26.7	25.6	27.5	22.6	22.6	22.6	15.8	15.6	16.1	28.8	28.8	28.7
20 - 35	65.8	66.3	65.5	73.4	73.3	73.4	73.8	74.5	72.9	64.2	64.1	64.3
> 35	7.4	8.1	7.0	4.0	4.1	4.0	10.4	10.0	10.9	7.0	7.1	7.0
No formal school %	9.7	9.6	9.8	2.7	2.8	2.6	18.1	16.6	20.5	2.0	2.0	1.9
Parity, %												
0	28.8	27.5	29.8	26.4	26.1	26.6	27.6	27.8	27.4	35.4	35.9	35.1
1	20.1	19.9	20.3	22.2	22.4	22.0	21.7	22.1	21.2	24.7	25.0	24.6
2+	51.1	52.6	49.9	51.5	51.5	51.4	50.6	50.1	51.4	39.7	39.1	40.4

^a^Denominators include missing values

Table 2 summarizes the indicators of care for each site during the trial. Rates of women having at least one antenatal care (ANC) visit ranged from 88.4 % in Pakistan to 100 % in Belgaum. The frequency of women having > 3 ANC visits ranged from 80.4 % of Guatemalan women to less than 20 % in Zambia. In the Indian sites, from 75 to 90 % of the women initiated ANC during the first trimester compared to less than 11 % of women initiating early ANC in the African sites. Around 50 % of the women in the Latin American sites initiated ANC in the first trimester. Physician delivery ranged from 74.6 % in Argentina to less than 3 % in both African sites. Women unattended during delivery, or attended by a family member only, ranged from <1 % in the Nagpur, Guatemalan and Argentinian sites to as many as 14 % in the African sites. Finally, 99.2 % of Argentina site women delivered in a hospital compared to 20 % or less in both African sites. Cesarean section rates ranged 2 % or less in the African sites to nearly 39 % in Argentina.

**Table 2:** Process of care by site and by intervention (Int) and control (Ctr) clusters

	South Asia											
	Belgaum			Nagpur			Pakistan					
	Total	Int	Ctr	Total	Int	Ctr	Total	Int	Ctr			
Deliveries, N	31,530	14,900	16,630	15,095	7,498	7,597	15,916	7,714	8,202			
Antenatal care (ANC) received, %	100.0	100.0	99.9	99.9	99.9	99.9	88.4	86.9	89.7			
>3 ANC visits	60.8	54.6	66.5	73.1	83.1	63.2	31.1	31.0	31.3			
ANC visit in 1st trimester	75.6	70.7	80.0	90.0	85.6	94.4	33.5	32.7	34.2			
Delivery attendant, %												
Physician	62.0	56.5	66.9	63.5	48.3	78.4	30.5	34.1	27.1			
Nurse/nurse midwife	33.6	39.3	28.5	35.2	50.4	20.2	26.3	24.9	27.7			
Traditional birth attendant	1.1	1.1	1.1	0.6	0.6	0.6	40.7	37.7	43.5			
Family/Unattended	3.4	3.2	3.6	0.7	0.6	0.7	2.5	3.3	1.7			
Delivery location, %												
Hospital	71.4	68.1	74.4	70.1	63.4	76.7	33.9	27.6	39.8			
Clinic	23.6	27.3	20.3	28.4	35.2	21.7	26.3	31.4	21.4			
Home/Other	5.0	4.6	5.3	1.4	1.3	1.6	39.8	40.9	38.8			
C-section, %	17.4	16.9	17.9	21.5	20.8	22.3	12.3	11.5	13.1			
	Sub-Saharan Africa						Latin America					
	Zambia			Kenya			Guatemala			Argentina		
	Total	Int	Ctr	Total	Int	Ctr	Total	Int	Ctr	Total	Int	Ctr
Deliveries, N	10,018	4,279	5,739	13,003	5,900	7,103	9,773	5,813	3,960	4,407	2,115	2,292
Antenatal care (ANC) received	99.7	99.7	99.6	98.8	98.9	98.7	99.0	98.8	99.2	95.5	96.6	94.6
> 3 ANC Visits	19.6	25.2	15.3	43.2	44.8	41.9	80.4	78.4	83.4	63.7	63.6	63.9
ANC in 1^st^ trimester	10.8	10.9	10.7	6.3	8.9	4.1	45.0	47.2	41.8	52.1	46.0	57.6
Delivery attendant, %												
Physician	2.1	1.4	2.5	2.5	3.0	2.1	46.7	47.8	45.1	74.6	68.1	80.7
Nurse/nurse midwife	63.4	67.6	60.3	44.9	49.5	41.1	1.5	2.2	0.5	24.9	31.6	18.8
Traditional birth attendant	20.3	16.7	23.0	38.9	31.6	44.9	51.4	49.7	53.9	0.0	0.0	0.0
Family/Unattended	14.2	14.3	14.1	13.7	15.9	11.9	0.5	0.4	0.6	0.4	0.3	0.5
Delivery location, %												
Hospital	20.1	26.8	15.1	15.2	16.8	13.9	43.9	43.6	44.3	99.2	99.1	99.3
Clinic	48.2	46.7	49.4	31.5	35.4	28.4	4.2	6.3	1.1	0.1	0.1	0.1
Home/Other	31.7	26.5	35.6	53.2	47.9	57.7	51.9	50.1	54.6	0.7	0.8	0.6
Cesarean section, %	1.0	0.9	1.2	2.0	2.3	1.7	20.6	21.3	19.6	38.6	35.9	41.1

The metrics of care varied by intervention and control cluster status for both antenatal care and delivery care. In Belgaum, India, measures of ANC tended to be better in the control clusters, while these measures were better in the intervention clusters in Nagpur, India. Few substantial differences in ANC between the intervention and control clusters were observed for other sites. In the Argentinian and both Indian sites, rates of physician-delivery were higher in the control clusters compared to the intervention clusters, while Pakistan and Guatemala had higher physician-delivery rates in the intervention clusters. Both African sites had low rates (≤3 %) of physician delivery, which were similar across intervention groups. With the exception of the two African sites which had lower rates of hospital births among interventions clusters, the sites generally had higher rates of hospital deliveries in the intervention clusters compared to the control clusters (though differences in Argentina were negligible). In regard to the rates of hospital delivery, similar trends between the intervention and control clusters were observed in the sites during the pre-trial period (data now shown).

Across all sites, the use of ACS was substantially higher in the intervention compared to the control clusters (Table 3).

**Table 3:** ACT intervention effect on antenatal corticosteroid use and 28-day neonatal mortality by site

Region	South Asia			Sub-Saharan Africa		Latin America	
Characteristic	Belgaum, India	Nagpur, India	Pakistan	Zambia	Kenya	Guatemala	Argentina
ACS use among all women %							
Intervention clusters	11.3	9.3	23.4	14.4	2.9	9.1	17.2
Control clusters	1.8	1.3	1.5	0.3	0.4	1.0	9.2
Neonatal mortality <28 days^a^, N (Rate/1000)							
Neonatal deaths <5^th^ %tile							
Intervention clusters	133 (249.5)	109 (305.3)	172 (226.3)	30 (151.5)	45 (191.5)	57 (164.7)	20 (219.8)
Control clusters	158 (255.7)	84 (329.4)	172 (250.4)	27 (127.4)	27 (142.9)	39 (234.9)	17 (129.8)
Neonatal deaths All births							
Intervention clusters	378 (25.8)	221 (29.8)	359 (48.4)	83 (19.6)	103 (17.6)	130 (22.6)	26 (12.4)
Control clusters	369 (22.5)	159 (21.3)	397 (49.8)	76 (13.3)	81 (11.5)	102 (26.1)	27 (11.8)
ACT intervention effect on neonatal mortality < 28 days^a^ [RR (95 % CI)^b^] for intervention versus control							
Neonatal deaths <5^th^ %tile	0.96 (0.75, 1.22)	0.94 (0.72, 1.23)	0.89 (0.80, 0.99)	1.43 (0.90, 2.28)	1.30 (0.94, 1.81)	0.75 (0.69, 0.82)	1.60 (0.99, 2.58)
Neonatal deaths All births	1.13 (0.99, 1.27)	1.36 (1.09, 1.71)	0.93 (0.82, 1.07)	1.77 (1.42, 2.20)	1.47 (1.02, 2.12)	0.86 (0.72, 1.03)	1.06 (0.54, 2.09)

^a^The denominator for neonatal deaths is live births

^b^Hypotheses test results: Relative Risk (RR) with corresponding 95 % CI and p-values calculated from generalized linear models with generalized estimating equations which account for the cluster-level variance and are adjusted for randomization strata

**Fig. 1:**
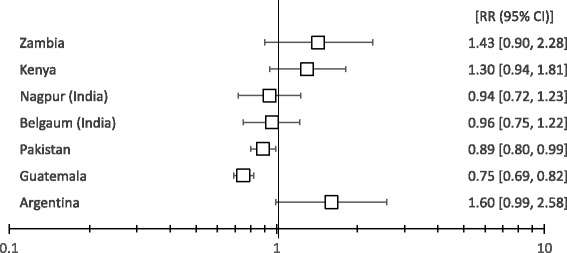
The RR of 28-day neonatal mortality among <5th percentile live births comparing the intervention to control clusters by site

**Fig. 2:**
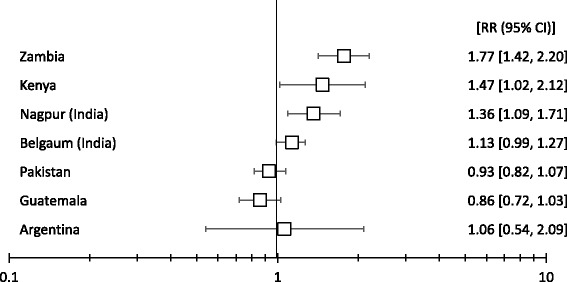
The RR of 28-day neonatal mortality comparing the intervention to control clusters among all births by site

The associations between being in the intervention clusters and 28-day neonatal mortality among the <5th percentile birth weight infants varied across sites. (Table 3, Fig. 1) Overall the intervention was associated with no benefit or harmful effect on all < 5^th^ percentile infants, but a reduction in neonatal mortality among the <5th percentile birth weight infants in Guatemala (RR 0.75; 95 % CI 0.69–0.82) as well as Pakistan (RR 0.89; 95 % CI 0.80–0.99) (Fig. 2). The experience of the Guatemala site, which showed the most favorable outcome, is described in detail elsewhere [Garces A, McClure EM, Figueroa L, et al.: A multi-faceted intervention including antenatal corticosteroids to reduce neonatal mortality associated with preterm birth: A case study from the Guatemalan Western Highlands, forthcoming].

For infants of all birth weights, across all sites the intervention compared to the control clusters was associated with a significant increase in the risk of neonatal death (RR 1 · 12, 95 % CI 1.02–1.22, *p* = 0.0127). When we stratified by site, this relationship was evident in the African sites: Zambia (RR 1.77; 95 % CI 1.42–2.20) and Kenya (RR 1.47; 95 % CI 1.02–2.12), and to a lesser extent in Nagpur, India (RR 1.36; 95 % CI 1.09–1.71). A marginally significant increase was also seen in Belgaum, India (RR 1.13; 95 % CI 0.99–1.27) (Table 3; Fig. 2). A significant association was not seen in the other sites.

**Fig. 3:**
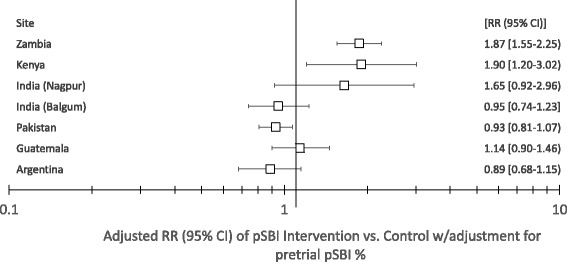
RR of pSBI comparing the intervention to control clusters among all births by site (adjusted for pretrial pSBI rates)

We assessed the effect of being in the intervention compared to the control clusters on pSBI, adjusting for the pre-trial pSBI rates of the clusters. Being in the intervention clusters was associated with a statistically significant increase in pSBI in the African sites, Zambia (RR: 1.87; 95 % CI: 1.55-2.25) and Kenya (RR 1.90; 95 % CI 1.20–3.02). pSBI was not significantly associated with the intervention for the other sites. (Fig. 3).

## Discussion

ACT is a large randomized trial to assess a program aimed at increasing ACS use among women at risk of preterm birth. The trial sites represented diverse LMIC settings across Latin America, Asia and sub-Saharan Africa. While there was no overall benefit to being in the intervention group across the entire study, sites in Guatemala and Pakistan had a significantly lower neonatal mortality in the intervention compared to the control clusters in the targeted (<5^th^ percentile) infants, while there was no evidence of benefit or harm in < 5^th^ percentile infants in the other sites.

Among all infants, the sites in Zambia, Kenya, and Belgaum and Nagpur (India) had a statistically or marginally significant increase in 28-day neonatal mortality in the intervention compared to the control clusters. The Zambian and Kenyan sites also had statistically significant increases in the risk of pSBI associated with the ACT intervention.

Considerable differences in ANC and obstetric care were found among the sites. Compared to the Asian and Latin American sites, the two sites in sub-Saharan Africa had substantially worse indicators of care. Few women delivered at a health facility, few deliveries were attended by a physician and the cesarean section rates were low.

Differences in care between the intervention and control clusters varied by site. While the clusters were stratified based on neonatal mortality rates prior to randomization, many of the differences in care observed between the intervention and control clusters appeared to precede the initiation of the ACT intervention. Overall, there were no clear patterns in the relationships between the outcomes and care when the intervention and control clusters were compared. For example, 28-day neonatal mortality was increased in the intervention clusters in Zambia despite increases in facility-based deliveries in those clusters. Similarly, the three sites with increased neonatal mortality had heterogeneous rates of skilled birth attendants at delivery. Kenya and Zambia had less care provided by traditional birth attendants or family birth attendants in the intervention compared to the control clusters, but an increase in overall neonatal mortality associated with the intervention. In the Pakistan site, where a slight benefit was associated with the intervention, no substantive differences in the processes of care were evident between the intervention and control clusters.

In contrast, in the Guatemala site, one of the two countries that showed positive effects associated with the intervention, care at delivery was better in the intervention compared to the control clusters including the use of ACS (which reached almost 50 % of the target group), delivery at a hospital or clinic, delivery by a physician and delivery by cesarean section. The findings in Guatemala are explored in detail elsewhere [Garces A, McClure EM, Figueroa L, et al.: A multi-faceted intervention including antenatal corticosteroids to reduce neonatal mortality associated with preterm birth: A case study from the Guatemalan Western Highlands, forthcoming].

The increased risk of pSBI in neonates associated with the intervention observed in the Kenyan and Zambian sites are consistent with the harmful effect on neonatal mortality also seen in the same countries. This finding is also in agreement with the overall increase in pSBI and death associated with the intervention reported in another paper of this series [Hibberd PL, Hansen NI, Wang M, et al.: Trends in the Incidence of Possible Severe Bacterial Infection and Case Fatality Rates in Rural communities in Sub-Saharan Africa, South Asia and Latin America, 2010–2013: A multicenter prospective cohort study, forthcoming]. Although these observations should be considered cautiously, they strengthen the plausibility that neonatal infection could have played a role in the observed harmful effect on neonatal mortality.

## Conclusions

Across all the sites there was no reduction in neonatal mortality in the <5^th^ percentile infants. However, Guatemala and to a lesser extent Pakistan had significant reductions in neonatal mortality in these infants. Guatemala, which had better indicators of care compared to some of the other sites and also in the intervention compared to the control clusters, showed benefit in the intervention compared to the control clusters in the targeted birth weight group. On the other hand, Kenya and Zambia showed a significant increase in neonatal mortality, as well as in suspected neonatal infection. Neonatal infection should be further investigated as a main outcome in future research studies.

These observations are intended as exploratory in nature and trends were not consistent across all sites. Because of the importance of identifying effective interventions to reduce neonatal mortality in the geographic areas with highest burden, further research is needed to define the impact of ACS across diverse settings in LMIC.
